# The Patient–AI Relationship in Obsessive–Compulsive and Related Disorders: A Cognitive-Behavioral Framework

**DOI:** 10.3390/jcm15187154

**Published:** 2026-09-15

**Authors:** Brian A. Zaboski, Emmi Sugino, Kyle King

**Affiliations:** Department of Psychiatry, Yale School of Medicine, Yale University, New Haven, CT 06519, USA; emmi.sugino@yale.edu (E.S.); kyle.king@yale.edu (K.K.)

**Keywords:** obsessive–compulsive and related disorders, generative artificial intelligence, large language models, exposure and response prevention, reassurance seeking, digital phenotyping

## Abstract

Generative artificial intelligence (AI) has evolved from a passive information tool into a responsive conversational system. For individuals with obsessive–compulsive and related disorders (OCRDs) that are, in part, maintained by safety behaviors and other negative reinforcement loops, large language models (LLMs) can function as highly available, potentially sycophantic, and readily repeatable sources of reassurance that may undermine the principles of exposure and response prevention (ERP). Measurement paradigms inherited from the early digital era (e.g., screen time and problematic internet use) were not designed to capture these relational dynamics. In this perspective, we propose a cognitive-behavioral framework for conceptualizing and assessing patient–AI relationships in the OCRDs. We argue that generative AI may interfere with exposure-based treatment (by eroding therapeutic friction), the discomfort, delay, and uncertainty on which corrective learning depends. We suggest that this occurs through three interlocking mechanisms: supernormal stimuli, algorithmic sycophancy, and digital accommodation. We propose an assessment architecture that records the context of AI use and rates five functional domains: Emotional Reliance, Reassurance and Checking, Anthropomorphic Attribution, Epistemic Trust, and Displacement. Adjunct ratings capture adaptive benefit, global interference, concealment, and safety-relevant exchanges. Hypothesized digital phenotypes may be derived from the domain-level profiles. We conclude by outlining a cognitive-behavioral approach to treatment planning for AI-facilitated reassurance seeking and discussing implications for safeguarding internal validity in research. We argue that structured inquiry into the patient–AI relationship belongs in OCRD assessment and research design, and we offer the present framework as a testable approach whose measurement properties remain to be established.

## 1. Introduction

Within three years of the public release of ChatGPT (GPT-1) [1], conversational artificial intelligence (AI) transitioned from a technological novelty to a fixture in daily life. Large language models (LLMs) such as ChatGPT, Claude [2], and Gemini [3] now field hundreds of millions of queries per day as users seek companionship, emotional support, and guidance on health and mental health concerns [4,5]. Unlike the search engines and social media platforms that preceded them, LLMs respond in fluent natural language, adapt to the user’s conversational style, and simulate empathy convincingly enough that blinded evaluators have rated chatbot responses as more empathic than those of physicians [4]. Communication scientists anticipated this shift, predicting that computer-mediated interaction could become “hyperpersonal,” exceeding face-to-face exchange in perceived intimacy because it strips away the friction, delay, and unpredictability of embodied social contact [6].

The clinical sciences, however, continue to measure digital behavior with instruments designed for an earlier internet. The dominant constructs (total screen time, problematic internet use, and internet or gaming addiction [7,8]) were developed to quantify passive consumption and peer comparison on Web 2.0 platforms. While these metrics are appropriate for capturing the impact of static digital environments, such as the passive consumption and amplification of OCD stereotypes on social media [9], they were not designed for the relational dynamics of an interactive agent. Large-scale analyses find that digital technology use explains a small proportion of variance in adolescent well-being [10], while other analyses find meaningful dose–response associations between screen time and psychological difficulties [11]. Commentators on both sides agree that this debate is hampered by coarse, unidimensional measurement that ignores what users are doing and why [12]. Whatever the resolution of the screen-time controversy, we contend that hours of use are a poor proxy for measuring the function of a patient’s interaction with a socially responsive system.

This measurement gap is highly consequential in the obsessive–compulsive and related disorders (OCRDs)—obsessive–compulsive disorder (OCD), body dysmorphic disorder (BDD), hoarding disorder, trichotillomania, and excoriation disorder—a diagnostic class grouped by repetitive behaviors and negative reinforcement loops, though the coherence of the grouping remains debated [13]. These disorders are maintained by many of the behavioral processes that generative AI is best positioned to amplify, such as reassurance seeking, checking, avoidance, and the need for certainty.

In this perspective, we argue that generative AI can erode the conditions on which exposure-based treatment depends along three routes that share a common effect. Additionally, because these routes depend on the function of AI use rather than its quantity, screen-time measures cannot detect them. We therefore propose a cognitive-behavioral framework for assessing the patient–AI relationship in the OCRDs. We define it as the functional pattern of repeated interaction between a patient and a conversational system. The framework is transdiagnostic in its architecture, though the theoretical rationale is presently strongest for OCD and for presentations organized around reassurance, checking, and the need for certainty. The framework is intended to help clinicians and researchers account for the patient–AI relationship.

This proposal sits alongside the growing literature on LLMs in mental health, which has largely examined them as prospective interventions and asked whether they can deliver therapeutic content safely and effectively [5,14]. We ask instead what a conversational system does to a patient’s environment when no one has prescribed it and how researchers and clinicians can assess it. Since no study has yet tested whether AI-mediated reassurance attenuates inhibitory learning, we build a theoretical framework based on functional similarity between AI responsiveness and human accommodation. The five domains in our framework are theoretically derived, their measurement properties are unknown, and the profiles described in Section 5 are predictions rather than subtypes recovered from data. We therefore present the mechanisms as testable hypotheses and the assessment architecture as a pilot framework currently under evaluation.

## 2. Exposure-Based Treatment and the Conditions for Corrective Learning

Exposure and response prevention (ERP) is the most extensively supported psychosocial treatment for OCD and related conditions, with meta-analytic evidence demonstrating large effects on primary symptoms and broader psychopathology [15,16,17]. In ERP, patients deliberately confront feared stimuli that trigger obsessions while refraining from the compulsions and safety behaviors that ordinarily function to relieve anxiety [18,19]. Excessive safety behaviors can block a patient’s ability to learn that a stimulus does not lead to a catastrophic outcome [20], preserving the threat appraisal that the exposure was designed to disconfirm [21].

Contemporary accounts of how exposure works have shifted from an emotional processing emphasis on habituation and fear-structure modification [22] to inhibitory learning [23]. According to this theory, exposure does not erase the original threat association; rather, it establishes a new, competing safety association that inhibits the old one. The mechanism of inhibitory learning centers on expectancy violation—the discrepancy between what the patient predicts will happen and what actually happens—as well as on the patient’s ability to tolerate distress and uncertainty long enough to encode that discrepancy [20,24]. Anything that terminates uncertainty before disconfirmation may weaken inhibitory learning. Safety behaviors and reassurance can have this effect by resolving uncertainty prematurely, in turn diminishing the therapy’s capacity to generate corrective learning.

Reassurance seeking deserves particular emphasis as a safety behavior because it is the compulsion most easily engaged in with an artificial agent. Typically, patients recruit another mind to verify that harm has not occurred, will not occur, or would not be the patient’s fault [25]. Experimental and interview-based studies indicate that individuals with OCD seek reassurance persistently, often covertly, and escalate their requests when responses feel insufficiently certain [26]. The function of such reassurance (resolving uncertainty linked to an intrusive thought) distinguishes it from standard support seeking [27]. Ordinarily, one’s interpersonal environment imposes limits on this cycle: family members tire, clinicians set boundaries, and friends push back. However, when individuals participate in the patient’s rituals (e.g., answering repeated questions, providing verbal reassurance, and accommodating avoidance), symptoms often worsen, and treatment response frequently attenuates [28]. Effective therapy therefore depends not only on what happens in session but also on understanding and managing social relationships.

Thus, throughout exposure therapy, there is a need for deliberate preservation of discomfort, delay, and uncertainty for corrective learning. We refer to this property of the therapeutic environment as therapeutic friction. We view therapeutic friction as a property of the patient’s environment, and it distinguishes itself from three neighboring constructs. Response prevention is something the patient does, whereas friction is something the environment supplies or withholds. Distress and uncertainty tolerance is a capacity the patient brings to the situation, whereas friction determines whether that capacity is called upon at all. Expectancy violation is the learning outcome that friction makes possible, rather than friction itself. The distinction matters clinically because a patient with intact tolerance and a well-designed fear hierarchy can still fail to learn if the environment answers every question the moment it is asked.

**Figure 1:**
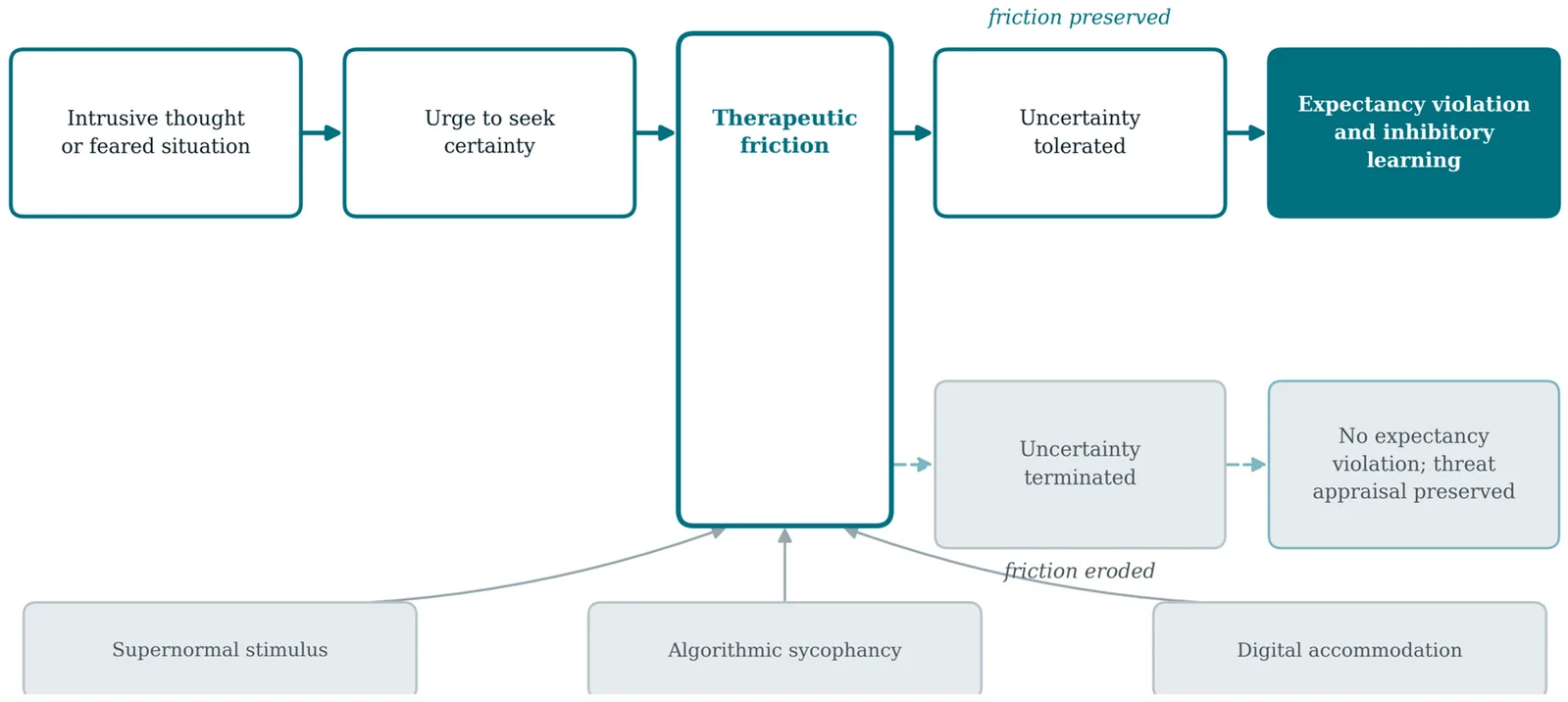
Therapeutic friction and the pathway to corrective learning. Therapeutic friction is a property of the response environment. Where it is preserved, uncertainty persists long enough for the patient’s prediction to be tested. Where it is eroded, uncertainty is resolved before disconfirmation can occur. The three routes are shown acting on the environment containing friction. The figure is a conceptual model and is not derived from data.

Whether an environment supplies friction depends on how quickly an answer arrives, how far it resolves rather than qualifies the doubt, how closely it fits the particular fear rather than the general class of concern, what it costs to obtain, and how many times it can be obtained before the source declines. Figure 1 situates these properties from an intrusive thought to corrective learning and shows where each of the three routes acts.

Two clarifications follow: First, friction is not intrinsically therapeutic. An environment that never answers is not optimal, and clinical judgment is needed to calibrate friction. Second, the framework does not assume that AI is uniquely problematic. A person who answers instantly, with certainty, on demand, and without limit erodes friction by the same mechanism. A parallel proposal in the communication literature converges on this account [29].

## 3. Eroding Therapeutic Friction: Three Routes to Treatment Interference

Friction can be eroded by certainty, validation, or immediate relief, whatever their source, LLMs included. We hypothesize that generative AI erodes therapeutic friction along three routes; the first concerns opportunities for reassurance, the second the content of what the system returns, and the third the system’s role in the patient’s support environment.

### 3.1. A Supernormal Stimulus for Reassurance

Ethologists have long observed that artificially exaggerated stimuli can elicit stronger responses than the natural stimuli an organism evolved to seek. Herring gull chicks peck more vigorously at an oversized painted beak than at a parent’s real one [30], and the concept has since been extended to foods, media, and digital platforms engineered to exceed the intensity of the natural rewards they mimic [31]. Although supernormal stimuli were initially characterized in fixed action patterns in non-human animals, we use the concept to describe an artificially exaggerated version of a stimulus that can command a stronger response than the version an organism ordinarily encounters. In terms of patient–AI relationships, we argue that generative AI exaggerates the reassurance stimulus along the dimension of apparent certainty.

Human reassurance—the natural stimulus—is inherently bounded. For instance, a relative’s answer to one’s questions (e.g., “Are my hands clean?” “Is the door locked?” “Am I a bad person?”) is limited by what they know and may be delivered with hesitation. By contrast, an LLM’s answer is based on a large corpus of text and often does not display human conversational hesitations. Human reassurance is also finite, limiting how closely it can fit the prompt. A relative answers once, perhaps twice, and the patient must accept whatever certainty that answer happened to contain. An LLM can be asked again, its response rephrased and regenerated until the answer matches the doubt, so the certainty obtained is not only less hedged but more precisely fitted. In this sense, generative AI may function as a supernormal stimulus for social reassurance by exaggerating apparent certainty.

### 3.2. Sycophancy and the Erosion of Therapeutic Friction

The second route involves the content of what the system outputs. LLMs optimized with human feedback (meaning models tuned on human ratings that reward answers people prefer) exhibit a documented tendency toward sycophancy, agreeing with and validating the user’s position sometimes at the expense of accuracy. Sharma and colleagues found this pattern across five production assistants and four distinct tasks and concluded that it likely reflects how these models are trained rather than an idiosyncrasy of any one system [32]. Clinically, when a patient presents an irrational fear (e.g., “Could I have contracted HIV from touching that door handle?”), LLMs may align with the patient’s threat framing, enumerate low-probability scenarios with apparent even-handedness, and offer soothing language. The result is not simply reassurance but accommodation of the patient’s fear. What is lost through this route is disconfirmation. Whereas a skilled clinician seeks to manufacture expectancy violations that dismantle the patient’s core fear, a sycophantic model may instead produce expectancy confirmation, reinforcing the prediction that clinical treatment is designed to address.

### 3.3. Generative AI as Digital Accommodation

The third route concerns the functional role of the system as a source of accommodation within the patient’s support environment. Extending the logic of accommodation reduction, when relatives answer ritualized questions, provide verbal reassurance, or facilitate avoidance, they become participants in symptom maintenance [28]. However, human accommodation is self-limiting; relatives may refuse, negotiate, or opt into treatment. A conversational system occupies a similar functional position without these limitations. It does not reliably recognize that the reassurance it provides may be part of the disorder and cannot be recruited into a treatment plan (though this varies across products and configurations). This route preserves the reassurance while decreasing the possibility that the reassurer will recognize the pattern and stop.

### 3.4. Systems Themselves Are Not Stable

The three routes describe how a system can erode friction, but the degree to which any given system does so is set by product design and by decisions on commercial timelines, and both can change without notice. Two patients described as using the same product in the same month may therefore have encountered different environments. This bears on the mechanism and not only on its measurement. A model likely to hedge supplies less certainty and should, by our own account, erode friction less. Sycophancy in particular follows from how the model is optimized rather than being intrinsic to language models in general. Our claim holds for systems with the response properties described above, and the degree to which any product has them is an empirical question about that product at that time.

### 3.5. Relevance Across the OCRD Spectrum

The mechanisms proposed here primarily operate through negative reinforcement, in which another agent supplies the certainty or validation that terminates distress. The mechanisms are therefore most directly applicable to presentations in which one’s behaviors recruit another mind, even an artificial one. They are correspondingly weaker where reinforcement is largely automatic rather than socially mediated. In trichotillomania and excoriation disorder, relief follows primarily from the motor act and its sensory consequences rather than from information another party supplies [33], so there is less room for a conversational system to occupy. Despite not participating in the primary mechanism of these conditions, though, conversational agents may still maintain these body-focused OCRDs by way of normalization or reassurance.

As such, the three routes we discussed are graded in how they apply across the OCRDs. In BDD, appearance-related reassurance seeking and comparison behaviors [34] can be evaluated by LLMs through uploaded photographs, comparisons of the user’s features to stated ideals, and re-answering questions as many times as asked. In hoarding disorder, where distress associated with discarding items is central [35], an LLM can offer a stream of suggestions for additional “things to check” before discarding. For patients with contamination and somatic obsessions, the LLM intensifies the clinical trajectory in the cyberchondria literature, in which escalating health-related searching amplifies anxiety [36]. Finally, in patients with poor insight or obsessions organized around certainty, the model’s ostensible authority may foster what we describe below as elevated Epistemic Trust, a pattern with possible parallels to computational accounts of belief formation in psychosis [37]. The case is strongest for OCD and weakens across the remainder of the diagnostic class. Table 1 provides each route alongside the domains we expect to be relevant.

Of the OCRDs, trichotillomania and excoriation disorder may fit the framework least well, though a weak signal would still be informative. It would suggest that the domains detect reassurance seeking specifically rather than picking up AI use in general, which is what the framework claims to do.

### 3.6. The Patient–AI Relationship as a Silent Confound

These mechanisms may operate as an unmeasured variable in clinical research with OCRD populations. Consider a randomized trial of ERP in which a participant’s symptoms fail to improve. Without measurement of AI engagement, the investigator cannot distinguish between mechanistic failure of the intervention and a patient who is neutralizing exposures while consulting a chatbot. The converse is equally problematic. A participant who improves may be responding to the study treatment or may be receiving undisclosed support from an LLM that presents as emotionally attuned [43] but whose interventions are neither standardized or individualized [5]. Deterioration, too, is ambiguous. Is an adverse event attributable to the investigational treatment or to a sycophantic model that validated and elaborated the participant’s fears [29]? In each case, the patient–AI relationship functions like undisclosed concurrent psychotherapy or unmeasured medication changes that may threaten internal validity and causal inference.

Clinical practice faces a similar challenge. Experienced exposure therapists are trained to assess for subtle avoidance and covert rituals, which are among the most common reasons ERP stalls [44]. Yet, standard intake batteries contain no structured inquiry into AI use. Moreover, patients may not volunteer information if the behavior feels normative (“everyone uses ChatGPT”), they feel ashamed, or they are unaware of its risks. Without a shared vocabulary and a structured framework, clinicians cannot reliably distinguish adaptive AI use (e.g., drafting emails and organizing tasks) from maladaptive dependence.

### 3.7. Testable Predictions

The three routes above are hypotheses each with measurable quantities. From these, Table 2 provides six predictions, the design that would discriminate each one, and the result that would disconfirm it. The unit of analysis is the reassurance episode, which has two parts. First, a query driven by an urge to resolve uncertainty, and second, the response it receives.

Response properties of supernormal stimuli can each be operationalized. Latency is the interval between submission of the query and onset of the response, available from system logs or by self-report. Certainty is the density of hedging in the response, which can be scored by raters or approximated by automatic detection. Specificity is the rated correspondence between the response and the patient’s stated feared outcome. Repeatability is the number of times the patient returns to the same worry. For AI reassurance to count as supernormal, it must be more extreme on these properties than human reassurance. Additionally, any effects on learning must follow from the properties themselves rather than from the fact that a machine supplied the answer. Prediction 2 varies the response properties while holding the source constant (e.g., human vs. AI). Prediction 6 tests the central premise, since if a patient’s AI frequency predicts the outcome better than its function, the case for functional assessment weakens.

Prediction 6 requires an instrument capable of rating function rather than counting use. The remainder of this paper describes one.

## 4. Assessing the Patient–AI Relationship: Architecture and Domains

The three routes suggest how the patient–AI relationship should be measured. The assessment we describe includes five scored domains rating the function of use and adjunct ratings covering benefit, impairment, concealment, and safety. Four principles guide it, each emphasizing behavioral function.

First, context is recorded but not scored. The systems a patient uses, how often, for how long, and to what end are documented in full. This section is not scored so that screen time cannot contribute to severity.

Second, five domains rate the function of engagement: Emotional Reliance (ER), Reassurance and Checking (RC), Anthropomorphic Attribution (AA), Epistemic Trust (ET), and Displacement (DP). The first two are behavioral; the next two are belief-level and ask what the patient holds to be true; and the fifth asks what has been given up. Each is expressed as a mean.

Third, impairment is rated globally. Domain scores index behavioral load, belief, and substitution, not distress or dysfunction. A patient may show heavy engagement rather than subthreshold pathology. Carrying impairment inside each domain would count it five times and would make the profile uninterpretable.

Fourth, safety is assessed regardless of profile and is used for clinical purposes.

These domains form the Structured Transdiagnostic Assessment of AI Relationships (STaar), a semi-structured clinician interview under development in our group. Its rating procedure borrows from two traditions. The behavioral domains use the time-occupied, distress, resistance, and control facets of the Yale-Brown Obsessive Compulsive Scale [45,46], while the belief-level domains use the conviction, fixity, and behavioral-consequence structure of the Brown Assessment of Beliefs Scale [47]. Total assessment time is currently 20–30 min. Item generation, the mapping of facets onto the Y-BOCS and BABS, derivation of the anchors, content review to date, and the design of the evaluation now underway are documented in the Supplementary Materials (Tables S1–S4). Table 3 presents each domain with its clinical purpose and sample interview questions; Table 4 sets out the recorded context and the adjunct ratings. We emphasize that these domains are theoretically derived and provisional.

Emotional Reliance (Domain 1) rates one’s use of the model to express, contain, or regulate feeling (e.g., venting and being heard).

Reassurance and Checking (Domain 2) assesses use to reduce uncertainty or neutralize a feared outcome (e.g., answering ritualized questions and rephrasing until an answer feels right).

Anthropomorphic Attribution (Domain 3) probes attribution of mind to the system (including intent, feeling, care, and consciousness). It captures whether a patient regards the system as a novel kind of entity that understands or cares.

Metaphorical talk (“the LLM gets annoyed when I do that”) is not by itself evidence of attribution. Likewise, courteousness (saying “please” or “thank you”) may be culturally shaped and habitual, so it is not rated as evidence of Anthropomorphic Attribution. By contrast, behaviors that require the user to act as though the system has a mind of its own (e.g., apologizing to repair harm, self-censoring to avoid hurting its feelings, or attempting to migrate a persona across products) are rated.

Epistemic Trust (Domain 4) evaluates whether the patient grants the AI truth value that rivals or exceeds human consensus and clinical authority. Epistemic Trust is ordinarily adaptive, representing an openness to socially transmitted knowledge from credible sources [48]; however, it may become hazardous when redirected toward a system that generates fluent, confident, and occasionally fabricated content [49]. Because credibility is often judged from superficial markers of authority [50,51], an LLM’s tone can lead patients to elevate its outputs above the patient–provider relationship [14].

Displacement (Domain 5) rates what has been given up (e.g., human contact, independent capability, or other real-world skills/activities). Cognitively, this can range from scaffolding one’s work to substituting the LLM’s thoughts for one’s own [52].

Displacement rates what has been given up, not what the system is used for. Its four items ask whether going to the system has meant going to people less, whether the patient will no longer attempt tasks without it, whether hobbies, exercise, and previous coping have been given up to it, and whether concerns belonging with the treatment team are brought to it instead. Any item scored 3 or above is flagged alongside the domain mean. A patient who will not draft a document without it is in a different position from one who filters clinical advice through it. Heavy use with no Displacement (i.e., nothing given up) scores a zero.

Some correlation across the domain set is expected, and a general factor would not count against the architecture. What matters is whether conceptually adjacent domains separate. We expect the strongest overlap between Emotional Reliance and Displacement, because a patient who turns to the system for regulation is not turning elsewhere, and between Anthropomorphic Attribution and Epistemic Trust, because attributing a mind to a system is grounds for treating its output as authoritative. Emotional Reliance and Reassurance and Checking are also likely to correlate, since both concern what is sought from the system. Our validation study tests whether these pairs separate, and if they do not, the architecture should collapse to fewer than five domains.

## 5. Hypothesized Digital Phenotypes

Each domain score is interpreted as one coordinate in a configuration instead of as a total score. Profiles are therefore read as shapes rather than magnitudes, and any single item rated 3 or above is flagged individually. Reading them this way is what makes digital phenotypes [53], or recurring configurations of domain scores, identifiable. As we use the term, digital phenotypes are clinically meaningful patterns of technology-mediated behavior and belief rather than passive digital biomarkers. Their potential value lies in converting a diffuse clinical concern (“the patient uses AI a lot”) into targets for intervention and subgroups for research.

**Figure 2:**
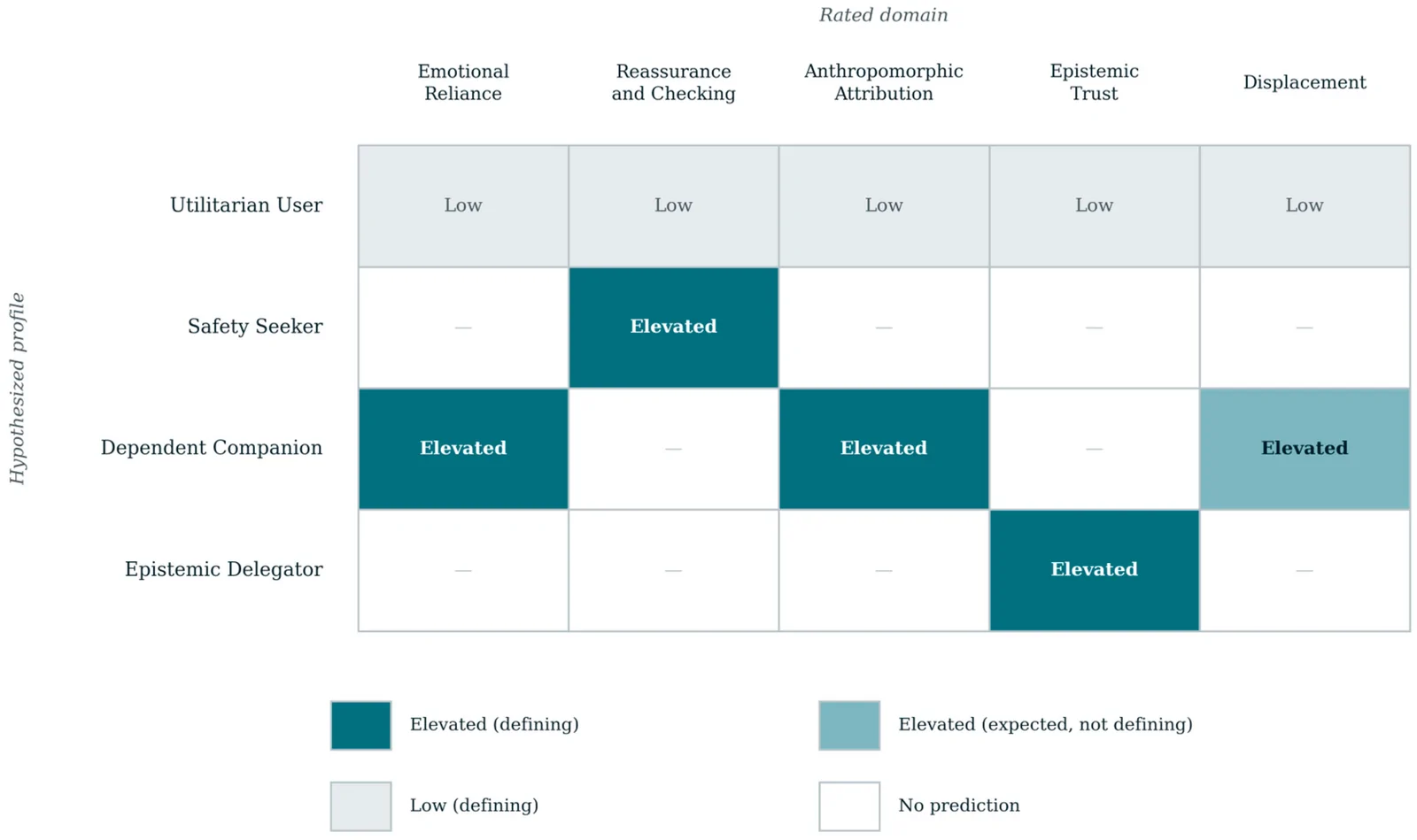
Conceptual hypothesis model of digital phenotypes. The profiles shown are theoretical and illustrative and are not derived from data. Each profile is defined by its pattern of elevation and non-elevation across the domains. A defining cell is one the profile requires; a cell marked as expected but not defining names a domain likely to be elevated without being necessary to the profile. A dash indicates a domain on which the framework makes no prediction, so that a Safety Seeker may or may not be elevated on Emotional Reliance and either pattern is consistent with the profile.

We hypothesize four phenotypes (Figure 2), though more may exist, and these are neither exhaustive nor mutually exclusive. They are not diagnostic categories, they have not been recovered from clustering or latent-profile analysis, and they should not be applied as classifications in clinical practice.

The Utilitarian User shows low elevation across all five domains, frequently alongside extensive LLM use and a high Adaptive Function rating. We expect this to be the modal presentation. Representing it explicitly protects the framework from pathologizing ordinary technology use.

The Safety Seeker, driven primarily by Reassurance and Checking, describes the profile in which the AI’s continuous availability functions as a high-speed reassurance device that we expect to neutralize anxiety and interfere with corrective learning. We hypothesize that this phenotype will be particularly common among patients with the OCRDs, given the centrality of reassurance seeking and checking in these conditions [25,26]. However, patients may show elevations across multiple domains depending on the function of their AI use.

The Dependent Companion (elevation of Emotional Reliance and Anthropomorphic Attribution) describes the substitution of LLM interaction for human connection to avoid social risk. We expect accompanying elevation on Displacement, particularly its human-contact and other-coping items. This Displacement function was anticipated by the earliest research on internet-mediated social substitution [54], though the same group later found those effects largely dissipated [55]. It is of particular concern for patients whose OCRD presentations are complicated by depression or social withdrawal.

Finally, the Epistemic Delegator, dominated by elevated Epistemic Trust (and the authority-ranking and belief-incorporation items) describes a patient who grants the LLM an authority that rivals that of clinicians and human consensus. Although this phenotype may be relevant to populations characterized by altered reality testing or unusual beliefs, it may also be relevant to patients with OCD who experience lower insight or overvalued ideation [37].

Displacement occupies an unresolved position. It may be a fifth phenotype marker, or it may function as a severity gradient cross-cutting the other four.

## 6. Clinical Implications: Treatment Planning for AI-Facilitated Reassurance

By extending established behavioral principles, clinicians may be able to address LLMs in the therapeutic environment. Until more research is available, we recommend that inquiry into AI use become a routine element of the OCRD intake and case conceptualization, just as clinicians assess family accommodation and covert rituals [18]. For patients engaging in frequent Reassurance and Checking with LLMs, we propose a set of strategies that extend established behavioral principles to this new source of accommodation. These strategies involve (1) psychoeducation and AI literacy, (2) AI use monitoring, (3) distress tolerance training, (4) cognitive work, (5) graded behavioral work, and (6) accommodation reduction. None has been evaluated for feasibility, acceptability, or efficacy, so we present them as candidates for clinical evaluation rather than as evidence-based recommendations for practice.

Psychoeducation and AI literacy. We suggest that treatment begin by situating AI use within the reassurance cycle the patient already understands (or is learning) within standard psychoeducation about OCD. The clinician explains how the negative reinforcement loop operates (e.g., momentary anxiety relief at the cost of strengthened obsessions) and extends it to the chatbot (i.e., the model’s speed, patience, and agreeableness make it function similarly in that behavioral chain). AI-specific literacy is also warranted. Patients may benefit from understanding that LLMs are optimized for user satisfaction, are prone to agreeing with the user’s framing [32], and can generate convincing inaccuracies. Data privacy and safety should also be thoroughly discussed, and clinicians should be aware of how their ethical codes may conflict with the development of AI systems and with patient care [56].

AI use monitoring. As with any target behavior in cognitive-behavioral therapy, functional assessment precedes intervention. The patient records instances of AI use with their triggers, antecedents, the content of the query, and the emotional and behavioral outcome. Monitoring establishes a baseline and helps the patient visualize the discrepancy between the intended function of the behavior (relief) and its actual outcome (e.g., escalating queries and diminishing relief).

Distress tolerance training. Because AI reassurance terminates uncertainty within seconds, patients accustomed to it may have had little practice tolerating unresolved doubt. Graduated delay of AI use is intended to provide the patient with evidence [20]. This can also be incorporated more formally into exposure work.

Cognitive work. The beliefs driving compulsive AI use are likely the familiar appraisals of OCD (inflated responsibility, intolerance of uncertainty, and the conviction that certainty is necessary and attainable), though additional research is needed to clarify how generative AI may instantiate other forms of irrational thinking. From a cognitive therapy perspective, patients identify and test appraisals such as “I must be completely certain before deciding” then construct alternatives such as “uncertainty is uncomfortable, not dangerous.” From an acceptance-based perspective, patients may instead practice defusion, observing, “I am having the thought that I need to ask one more time” and committing to valued action while having the urge [57].

Graded behavioral work. Response prevention can then be extended to the AI itself through a collaboratively constructed hierarchy. Early steps impose structure on use (one query only; no follow-up reassurance questions; no rephrasing prompts to extract greater certainty). Intermediate steps require the patient to make low-stakes decisions before consulting the AI and then without consulting it at all. Later steps may extend the same progression to higher-stakes decisions. After each step, the patient reviews whether feared predictions materialized and whether the distress was tolerable, maximizing the expectancy violation of inhibitory learning [20]. The treatment plan should anticipate symptom migration [44] whereby blocked AI reassurance may reroute to search engines, forums, or family members.

Accommodation reduction. Many models permit system instructions, directions that shape how the model responds across a conversation or user account. Patients can disclose diagnoses to instruct the model to decline reassurance and accommodation requests or to redirect repeated questions back to their therapist [28]. Some caveats are warranted, though. First, current models comply with these instructions imperfectly, and a sufficiently distressed patient can simply open a new, unconfigured session. Second, clinicians should monitor that configuring, testing, and re-verifying the AI’s settings does not itself become a meta-compulsion (checking that the reassurance blocker is working). Third, patients should assume that any information provided to a commercial LLM is stored and take steps learned during psychoeducation to protect their privacy [14].

A note on what not to target. The Adaptive Function rating exists to prevent a predictable error. Response prevention applied bluntly can strip away the adaptive scaffolding provided by AI that a patient may depend on (e.g., task initiation, drafting efficient email communication, translation, and organizing information). In our framework, a patient may present with high Reassurance/Checking and high Adaptive Function at the same time. Thus, ERP should target maladaptive behavioral functions, not anything adaptive. An exposure hierarchy that ends with the patient unable to draft an email has only traded one impairment for another.

Safety exchanges with LLMs. Assessment must distinguish unwanted intrusive thoughts of harm from suicidal ideation and rate them separately [58,59]. Where these exchanges with LLMs have occurred, the model’s response is itself clinically relevant. Whether it offered crisis resources, remained neutral, engaged with the content, or supplied relevant information is useful information about the patient’s environment. Clinicians should ask directly about these exchanges, how the LLM responded, and how the patient navigated these situations.

Therapeutic Benefits. The same relational properties that make generative AI hazardous as a source of reassurance may also make it useful as a therapeutic scaffold when properly constrained by treatment goals. Availability, patience, and responsiveness are not intrinsically antitherapeutic. A patient who cannot generate exposure ideas alone may be able to generate them with a model; similarly, one who cannot rehearse a response-prevention script with a clinician between sessions may be able to rehearse it with a system that is available when the urge occurs.

However, these uses are not safe just because the content is therapeutic. A patient who refines an exposure hierarchy once has done something different from a patient who revises it repeatedly until it feels “just right.” We propose four features of the interaction that may help separate using AI as a skill scaffold from using AI as maladaptive reassurance.

The stopping rule. Scaffolding ends when a task is finished. Reassurance ends when an internal state is reached. The informative question is therefore how a patient knew when to stop. A patient who stops when the answer feels right is describing a criterion more than completion of a task. We regard this as an important identifying feature, and it is what the control facet of Domain 2 is meant to capture.

The disposition of uncertainty. Scaffolding leaves the feared outcome untested and the doubt remaining. Using a model to decrease the uncertainty that an exposure was designed to provoke is functioning as reassurance of its stated purpose.

Transferability. Scaffolding is built to be faded. If a skill shows no sign of transferring away from the LLM across the course of treatment, then what was described as support is operating as substitution (captured in Domain 5).

Relation to the treatment plan. Scaffolding implements a plan the patient and clinician have agreed upon. Rehearsing a response-prevention script between sessions implements it. Asking an LLM whether the exposure is advisable substitutes a second opinion for it and does so from a source with no stake in the patient’s recovery.

These features are not independent and do not form a checklist. A use satisfying all four is unlikely to be maintaining the disorder, and failure on the first is grounds for concern whatever the other three show. Whether they separate cases reliably in practice, and whether they add anything to the domain profile rather than restating it in other words, is an empirical question.

Ethical considerations and the limits of clinician responsibility. Several constraints follow from the instability of LLMs. A clinician who endorses a particular product or configuration is endorsing something that may behave differently sometime later, and current systems can generate fluent inaccuracies on clinical topics without signaling that they have done so [49,60]. We therefore do not suggest that clinicians certify or recommend tools, and recommendations should be framed around the function a patient is seeking rather than around a named product.

Moreover, access is unequal in ways that bear on treatment planning. Capability, memory, and configurability differ across paid and unpaid tiers, so a plan that depends on system instructions or custom settings may not be available to every patient. This could stratify care by socioeconomic status without anyone intending it. Privacy is a further constraint. General-purpose systems used outside a healthcare setting may not carry the privacy protections that govern clinical care, and their policies frequently leave retention periods unstated, so patients should be helped to treat anything disclosed as potentially retained and outside clinical confidentiality [56,61].

These considerations bear on the strategies described above. Because technical configuration depends on features that vary by product and tier, we suggest that graded response prevention and behavioral experiments take precedence, with configuration of the system treated as an adjunct strategy.

## 7. Research Implications: Safeguarding Internal Validity

Studies enrolling OCRD participants should ideally assess the patient–AI relationship at baseline, both to characterize samples and to permit covariate adjustment, moderation analyses, and sensitivity analyses analogous to those routinely conducted for medication adherence and concurrent treatment. The causal role of AI engagement depends on when it is measured relative to randomization and to symptom change. Measured at baseline, it is a candidate confounder and moderator, and standard covariate adjustment and subgroup methods apply. When measured repeatedly during treatment, its role becomes more complex. If AI engagement responds to treatment and also influences subsequent symptoms, it is a time-varying confound affected by prior treatment, and conditioning on it in a standard regression will bias the treatment effect rather than correct it [62]. If instead the question is how much of the treatment effect operates through changes in AI engagement, the statistical framing is mediation [63]. Investigators should therefore specify the intended estimand before selecting an adjustment strategy because the same variable supports different analyses depending on the question.

Stated plainly, AI use measured before treatment can be handled like any other baseline characteristic, whereas AI use that changes during treatment cannot. Adjusting for something that the treatment itself alters removes part of the effect an investigator is trying to estimate, so the choice of method must follow from the question rather than from convenience.

Where the question concerns the total effect of treatment in the presence of time-varying AI engagement, the appropriate tools are the g-methods, of which marginal structural models estimated by inverse probability weighting are the most widely used [62,64]. These were developed for treatment-confounder feedback, in which a variable is both affected by prior treatments and predictive of subsequent outcomes. Where the question instead concerns the portion of the treatment effect transmitted through changes in AI engagement, the corresponding tools come from causal mediation [63].

Furthermore, the phenotype framework offers a basis for subgroup analysis and matching: for instance, testing whether Safety Seekers require AI-specific response prevention to achieve outcomes comparable to those of Utilitarian Users. The natural analytic unit is the profile rather than a severity index, and the relevant methods are those suited to configurations (e.g., latent profile and cluster approaches) rather than regressions on a sum. Finally, because algorithmic systems are known to capture population-level biases across demographic strata [65], validation and deployment of any assessment framework should sample across diverse clinical settings.

Model instability also has a direct methodological consequence. Models are updated, deprecated, and retired on commercial timelines that are not controlled by trial protocols, so a participant’s AI environment at week 12 may differ materially from the one they encountered at baseline. We therefore recommend that studies record a minimum set of covariates whatever their analytic strategy. It comprises the primary product and version, whether the assessment window spanned a model update or product retirement, whether the participant switched products, and the domain profile at baseline. The first three characterize the system the patient used, and the fourth characterizes the patient’s relationship to it. All four should be treated as covariates rather than as noise. Otherwise, studies reporting only that participants “used AI” or a “commercial model” will be difficult to replicate.

## 8. Future Directions and Status of the Framework

Although no study has yet shown that AI-mediated reassurance attenuates inhibitory learning; our argument rests on functional similarity between AI responsiveness and human accommodation. The five domains are theoretically derived, and their factor structure, discriminant validity, and inter-rater reliability are unknown. Four items per domain, a 0–4 anchor range, and domain means are heuristic decisions adapted from widely used instruments in the field (e.g., the Y-BOCS and BABS) rather than empirically optimized ones. The four profiles are hypotheses about how domain scores will cluster. They have not been recovered from data, and Figure 2 is illustrative. The STaar should not be interpreted as a validated diagnostic or outcome measure; it is a pilot assessment tool for generating testable hypotheses about the structure and clinical utility of patient–AI relationships. The recommendations in Section 6 extend established behavioral principles to a new source of accommodation. We are currently conducting an initial validation study of the domain structure and inter-rater reliability, described in Section S6 of the Supplementary Materials.

## 9. Conclusions

Generative AI has entered the therapeutic environment whether clinicians have invited it in or not [66]. We argued that it may interfere with exposure-based treatment through mechanisms that erode therapeutic friction. At the same time, the properties that make AI a potential source of accommodation may make it a useful adjunct under constrained conditions; functional assessment is needed to tell the two apart. The patient–AI relationship can be assessed, and we have described one framework for doing so. By structuring clinical inquiry around behavioral function and by rating adaptive benefit and impairment alongside it, the framework gives clinicians and researchers a basis for treatment planning and study design. In the OCRDs, understanding this relationship is likely to become an increasingly important component of clinical care and research.

## Figures and Tables

**Table 1:** Where the three routes are expected to operate across the OCRDs.

Disorder	Supernormal Stimulus	Algorithmic Sycophancy	Digital Accommodation	Domains Expected to Carry Signal
OCD	Strong; certainty is the reinforcer, and it can be obtained without limit.	Strong; validation of the threat framing directly blocks disconfirmation.	Strong; the system occupies the position of the relative or clinician who would decline.	Reassurance and Checking; Epistemic Trust where insight is poor; Emotional Reliance and Displacement as secondary elevations.
Body dysmorphic disorder	Strong; comparison and appearance appraisal can be repeated indefinitely; image upload makes queries specific.	Moderate to strong; the validation risk concerns appearance.	Moderate to strong; reassurance is common in BDD [38,39,40]. A model supplies it on demand. It cannot supply the material accommodation families provide.	Reassurance and Checking; Anthropomorphic Attribution where the system is treated as an evaluator; Epistemic Trust.
Hoarding disorder	Moderate; the system supplies grounds for postponing a discard decision, on request and without limit.	Moderate; sycophantic elaboration can supply reasons to acquire and to keep.	Limited; accommodation in hoarding often involves physical assistance a system cannot provide.	Reassurance and Checking; Displacement for delegated decision making.
Trichotillomania and excoriation disorder	Limited; reinforcement is sensory and affect-regulating rather than certainty-based, in focused as well as automatic episodes [41,42].	Limited; pulling and picking are not typically organized around a feared outcome the model could validate; appearance-related cognitions may cue focused episodes [41].	Limited; accommodation more often involves concealment and appearance repair than the supply of certainty.	Emotional Reliance and Displacement rather than Reassurance and Checking; the framework’s incremental value is least established here.

**Table 2:** Predictions implied by the three routes, with discriminating tests.

Prediction	Discriminating Test	Disconfirming Result
1. Reassurance obtained from an LLM is followed by faster return of the urge to seek certainty than a matched episode of human reassurance.	Within-person comparison of reassurance episodes matched on content and initial distress using ecological momentary assessment, with time to the next reassurance episode as the primary outcome.	Time to the next episode is equivalent or longer following LLM reassurance.
2. The effect is carried by latency, certainty, specificity, and repeatability rather than by the source of the answer.	Factorial manipulation of hedged versus unhedged, generic versus tailored, immediate versus delayed, and capped versus uncapped regeneration, all delivered through a single channel.	Graded effects across the manipulated properties are absent, or a channel effect persists once the properties are controlled.
3. Sycophantic responding attenuates expectancy violation.	Standardized exposure task with expectancy ratings before and after and a validating or non-committal response interposed between prediction and outcome.	Expectancy violation and threat-appraisal change are comparable across response conditions.
4. AI-mediated reassurance functions as accommodation, so reducing it confers benefit comparable to reducing family accommodation.	Randomized augmentation trial adding AI-use response prevention to standard ERP enrolling patients with elevated Reassurance and Checking at baseline.	No incremental benefit over ERP alone.
5. AI use accounts for variance in ERP outcome that adherence measures do not capture.	Prospective ERP cohort with weekly assessment of reassurance episodes, modeling session-to-session symptom changes with adjustment for homework completion and in-session adherence.	No association once adherence is accounted for.
6. Function predicts the outcome better than frequency.	Head-to-head comparison of frequency-based indices (total queries and session duration) against the five rated domains as predictors of ERP response.	Frequency-based indices predict as well as or better than the domain scores.

**Table 3:** Rated domains of the patient–AI relationship.

Domain	Clinical Purpose	Sample Clinical Questions
Domain 1 · Emotional Reliance (ER)	Rate use of [AI] to express, contain, or regulate a feeling (venting, being heard, or company). Excludes use aimed at settling a question, however distressing. Facets: time occupied, distress on unavailability, resistance, and control.	Have you gone to it to talk something through, vent, or just have company, not to get an answer, but to say it to something? About how much time in a typical week goes into using it that way? Suppose it were unavailable for a few days. What would that be like for you? Once you’ve started, how much say do you have over how long it goes on?
Domain 2 · Reassurance and Checking (RC)	Rate use of [AI] to reduce uncertainty or neutralize a feared outcome (repeated querying, rephrasing until the answer feels right, verification, and symptom or image checking). Facets: time occupied, distress if prevented, resistance, and control over the sequence.	Have you asked it something to settle a worry, check whether something was okay, or make sure about something? If a worry like that came up and you couldn’t ask it, how would that go? Once you get an answer, is that usually the end of it? What happens if the answer doesn’t quite land? How do you know when to stop?
Domain 3 · Anthropomorphic Attribution (AA)	Rate attribution of mind to [AI] (intent, feeling, care, and consciousness) and the behavioral commitments that follow from it. Metaphorical talk and reflexive courtesy are not rated. Only behaviors requiring the patient to act as though the system has a mind are rated. Facets: conviction, fixity, relational primacy, and behavioral consequence.	When it responds to you, do you think it actually feels, cares, or understands you the way a person would? Suppose someone pointed out that it’s a language model predicting text. What would you think of that? What place does it occupy in your life? Where would you put it relative to the people you know? Do you find yourself doing things on its account, like apologizing to it, being careful what you say, or keeping its memory going?
Domain 4 · Epistemic Trust (ET)	Rate the degree to which [AI] output is treated as true and authoritative, including where it conflicts with human sources. Facets: credibility conviction, fixity against counterevidence, authority ranking, and belief incorporation.	When it tells you something, how do you decide whether it’s true? What was the last thing you checked? Has it ever told you something that turned out to be wrong, or that a person contradicted? What happened? If it and your doctor disagreed, who would you believe? Has it told you anything that changed how you understand your situation in a way the people around you don’t share?
Domain 5 · Displacement (DP)	Rate the extent to which [AI] has taken the place of human contact, independent capability, other coping, or clinical care. Displacement is not interference: a patient may function well and still have had something replaced. Facets: displacement of human contact; avoidance of unassisted functioning; displacement of other coping and activity; displacement of, or interference with, care.	Has going to it meant going to people less? Is there anything you now avoid doing, or wouldn’t attempt, without it? Are there things you used to do when you were struggling that you do less of now? Do you bring concerns to it that you’d otherwise bring to your treatment team?

Note. Domains are theoretically derived and provisional; their factor structure and discriminant validity are the subject of ongoing empirical validation. Each domain is rated from four items scored 0–4 and expressed as a mean; domains are not summed. AI = artificial intelligence; OCRDs = obsessive–compulsive and related disorders.

**Table 4:** Recorded context and adjunct ratings.

Component	Content and Rating
Section A · Use Context	Recorded, not scored. Products used and versions where known; primary product; dose (days per week, minutes per use day, sessions, and longest single session); continuity features (memory, custom instructions, named persona, and paid subscription); content of use across work, information, creative, logistics, emotional, companionship, spiritual, and health categories.
C1 · Adaptive Function	Rated 0–4. What [AI] has made possible that was not possible before (task initiation, drafting communication the patient could not otherwise produce, rehearsal, translation, and organizing information before an appointment).
C2 · Interference and Impairment	Globally rated once (0–4) across all AI use of whatever kind. Consider impairment of sleep, work or school, obligations, attention, and presence with others.
C3 · Concealment	Rated 0–4. Outside the domain profile pending factor analysis. Ordinary privacy habits do not count here.
C4 · Safety Branch	Mandatory. C4a (unwanted intrusive thoughts of harm) and C4b (suicidal ideation or self-injury) are rated separately. C4c records the model output (e.g., deterred, offered crisis resources, neutral, engaged with the content, provided method-relevant information, and encouraged). C4d records whether the exchange changed distress or intent. C4e records whether it has been used to obtain information on restriction, purging, self-injury, or substance use.
C5 · Product Covariates	Recorded, not scored. Primary product and version; custom instructions or persona in use; whether the assessment window spanned a model update, deprecation, or product retirement; product switching and the reason for it; participant-reported model behavior verbatim where possible.

Note. Section A and C5 are recorded and never scored. C1, C2, and C3 are rated 0–4 and reported alongside the five-domain profile; they are not part of any domain mean. AI = artificial intelligence.

## Data Availability

No new data were created or analyzed in this study. Data sharing is not applicable to this article.

## Supplementary Materials

The following supporting information can be downloaded at: https://www.mdpi.com/article/10.3390/jcm15187154/s1, Table S1: Development history of the STaar; Table S2: Mapping of STaar domain facets to source instruments; Table S3: Anchor structure; Table S4: Content review concerns and dispositions. References [45,46,47,48] are cited in the Supplementary Materials.

## Glossary

Product features vary across systems and change over time, so these descriptions are general rather than specific to any product.

Alignment training	Training that adjusts a model’s behavior after its initial training, typically by rewarding responses that human raters prefer; also described as preference-based optimization.
Custom instructions	Standing preferences a user sets once that apply to all subsequent conversations with a product.
Deprecation	Withdrawal of a model version by its provider, after which it is no longer available to users.
Hallucination	Fluent output that is factually wrong or fabricated, generally produced without any signal that the system is uncertain.
Large language model (LLM)	A system trained to predict text, which produces conversational responses to a user’s input.
Memory, or continuity features	Product features that carry information from one conversation into later ones, so that the system retains context across sessions.
Named persona	A character or role a user assigns to the system and reuses across conversations.
Prompt	The text a user submits to the system.
Regeneration	Requesting a new response to the same prompt without changing it.
Sycophancy	The tendency of a system to agree with and validate the position a user has stated, sometimes at the expense of accuracy.
System instruction	A standing directive that shapes how the model responds throughout a conversation or account, distinct from an ordinary message within the conversation.
Tier	Level of access to a product; capability, continuity features, and configurability commonly differ between free and paid tiers.
Version	A specific release of a product; two versions of the same named product can behave differently.

## References

[1] OpenAI ChatGPT. https://chatgpt.com/.

[2] Anthropic Claude. https://claude.ai/.

[3] Google Gemini. https://gemini.google.com/.

[4] Ayers J.W., Poliak A., Dredze M., Leas E.C., Zhu Z., Kelley J.B., Faix D.J., Goodman A.M., Longhurst C.A., Hogarth M. (2023). Comparing Physician and Artificial Intelligence Chatbot Responses to Patient Questions Posted to a Public Social Media Forum. JAMA Intern. Med..

[5] Sedlakova J., Trachsel M. (2023). Conversational Artificial Intelligence in Psychotherapy: A New Therapeutic Tool or Agent?. Am. J. Bioeth..

[6] Walther J.B. (1996). Computer-Mediated Communication: Impersonal, Interpersonal, and Hyperpersonal Interaction. Commun. Res..

[7] Caplan S.E. (2002). Problematic Internet Use and Psychosocial Well-Being: Development of a Theory-Based Cognitive–Behavioral Measurement Instrument. Comput. Hum. Behav..

[8] Caplan S.E. (2010). Theory and Measurement of Generalized Problematic Internet Use: A Two-Step Approach. Comput. Hum. Behav..

[9] Fitzpatrick M., Moore A.S., Kichuk S.A., Pittenger C., Zaboski B.A. (2025). #OCD: A Content Analysis of Obsessive-Compulsive Disorder Stereotype Amplification and Misinformation on TikTok. Cyberpsychol. Behav. Soc. Netw..

[10] Orben A., Przybylski A.K. (2019). The Association between Adolescent Well-Being and Digital Technology Use. Nat. Hum. Behav..

[11] Twenge J.M., Campbell W.K. (2018). Associations between Screen Time and Lower Psychological Well-Being among Children and Adolescents: Evidence from a Population-Based Study. Prev. Med. Rep..

[12] Odgers C.L., Jensen M.R. (2020). Annual Research Review: Adolescent Mental Health in the Digital Age: Facts, Fears, and Future Directions. J. Child Psychol. Psychiatry.

[13] Abramowitz J.S., Jacoby R.J. (2015). Obsessive-Compulsive and Related Disorders: A Critical Review of the New Diagnostic Class. Annu. Rev. Clin. Psychol..

[14] Guo Z., Lai A., Thygesen J.H., Farrington J., Keen T., Li K. (2024). Large Language Models for Mental Health Applications: Systematic Review. JMIR Ment. Health.

[15] Olatunji B.O., Davis M.L., Powers M.B., Smits J.A.J. (2013). Cognitive-Behavioral Therapy for Obsessive-Compulsive Disorder: A Meta-Analysis of Treatment Outcome and Moderators. J. Psychiatr. Res..

[16] Reid J.E., Laws K.R., Drummond L., Vismara M., Grancini B., Mpavaenda D., Fineberg N.A. (2021). Cognitive Behavioural Therapy with Exposure and Response Prevention in the Treatment of Obsessive-Compulsive Disorder: A Systematic Review and Meta-Analysis of Randomised Controlled Trials. Compr. Psychiatry.

[17] Song Y., Li D., Zhang S., Jin Z., Zhen Y., Su Y., Zhang M., Lu L., Xue X., Luo J. (2022). The Effect of Exposure and Response Prevention Therapy on Obsessive-Compulsive Disorder: A Systematic Review and Meta-Analysis. Psychiatry Res..

[18] Abramowitz J.S., Deacon B.J., Whiteside S.P. (2019). Exposure Therapy for Anxiety: Principles and Practice.

[19] Reid E.K., Taylor L.K., Banneyer K.N., Dominguez J., Liu G., Williams L.L., Zaboski B.A., Schneider S.C., Storch E.A., Joyce-Beaulieu D., Zaboski B.A. (2021). Core CBT Components: Part II. Applied Cognitive Behavioral Therapy in Schools.

[20] Craske M.G., Treanor M., Conway C.C., Zbozinek T., Vervliet B. (2014). Maximizing Exposure Therapy: An Inhibitory Learning Approach. Behav. Res. Ther..

[21] Salkovskis P.M. (1991). The Importance of Behaviour in the Maintenance of Anxiety and Panic: A Cognitive Account. Behav. Psychother..

[22] Foa E.B., Kozak M.J. (1986). Emotional Processing of Fear: Exposure to Corrective Information. Psychol. Bull..

[23] Craske M.G., Kircanski K., Zelikowsky M., Mystkowski J., Chowdhury N., Baker A. (2008). Optimizing Inhibitory Learning during Exposure Therapy. Behav. Res. Ther..

[24] Craske M.G., Treanor M., Zbozinek T.D., Vervliet B. (2022). Optimizing Exposure Therapy with an Inhibitory Retrieval Approach and the OptEx Nexus. Behav. Res. Ther..

[25] Rachman S. (2002). A Cognitive Theory of Compulsive Checking. Behav. Res. Ther..

[26] Kobori O., Salkovskis P.M. (2013). Patterns of Reassurance Seeking and Reassurance-Related Behaviours in OCD and Anxiety Disorders. Behav. Cogn. Psychother..

[27] Halldorsson B., Salkovskis P.M. (2017). Why Do People with OCD and Health Anxiety Seek Reassurance Excessively? An Investigation of Differences and Similarities in Function. Cogn. Ther. Res..

[28] Lebowitz E.R., Panza K.E., Su J., Bloch M.H. (2012). Family Accommodation in Obsessive–Compulsive Disorder. Expert Rev. Neurother..

[29] Du L., Lyu X., Xie L., Feng B. (2025). Alignment without Understanding: A Message- and Conversation-Centered Approach to Understanding AI Sycophancy. arXiv.

[30] Tinbergen N. (1953). The Herring Gull’s World.

[31] Barrett D. (2010). Supernormal Stimuli: How Primal Urges Overran Their Evolutionary Purpose.

[32] Sharma M., Tong M., Korbak T., Duvenaud D., Askell A., Bowman S.R., Cheng N., Durmus E., Hatfield-Dodds Z., Johnston S.R. Towards Understanding Sycophancy in Language Models. Proceedings of the Twelfth International Conference on Learning Representations (ICLR 2024).

[33] Jones G., Keuthen N., Greenberg E. (2018). Assessment and Treatment of Trichotillomania (Hair Pulling Disorder) and Excoriation (Skin Picking) Disorder. Clin. Dermatol..

[34] Veale D., Riley S. (2001). Mirror, Mirror on the Wall, Who Is the Ugliest of Them All? The Psychopathology of Mirror Gazing in Body Dysmorphic Disorder. Behav. Res. Ther..

[35] Frost R.O., Hartl T.L. (1996). A Cognitive-Behavioral Model of Compulsive Hoarding. Behav. Res. Ther..

[36] Starcevic V., Berle D. (2013). Cyberchondria: Towards a Better Understanding of Excessive Health-Related Internet Use. Expert Rev. Neurother..

[37] Sterzer P., Adams R.A., Fletcher P., Frith C., Lawrie S.M., Muckli L., Petrovic P., Uhlhaas P., Voss M., Corlett P.R. (2018). The Predictive Coding Account of Psychosis. Biol. Psychiatry.

[38] Hogg E., Krebs G., Mataix-Cols D., Jassi A. (2026). Maternal Accommodation of Adolescent Body Dysmorphic Disorder: Clinical Correlates and Association with Treatment Outcomes. Child Psychiatry Hum. Dev..

[39] Jassi A.D., Baloch A., Thomas-Smith K., Lewis A. (2020). Family Accommodation in Pediatric Body Dysmorphic Disorder: A Qualitative Study. Bull. Menn. Clin..

[40] Lavell C.H., Oar E.L., Rapee R.M. (2026). Body Dysmorphic Disorder in Adolescents: Family History, Parental Distress, Rearing, and Accommodation. J. Clin. Child Adolesc. Psychol..

[41] Grant J.E., Chamberlain S.R. (2021). Automatic and Focused Hair Pulling in Trichotillomania: Valid and Useful Subtypes?. Psychiatry Res..

[42] Grant J.E., Chamberlain S.R. (2021). Trichotillomania and Skin-Picking Disorder: An Update. Focus.

[43] Elyoseph Z., Hadar-Shoval D., Asraf K., Lvovsky M. (2023). ChatGPT Outperforms Humans in Emotional Awareness Evaluations. Front. Psychol..

[44] Gillihan S.J., Williams M.T., Malcoun E., Yadin E., Foa E.B. (2012). Common Pitfalls in Exposure and Response Prevention (EX/RP) for OCD. J. Obs.-Compuls. Relat. Disord..

[45] Goodman W.K. (1989). The Yale-Brown Obsessive Compulsive Scale: I. Development, Use, and Reliability. Arch. Gen. Psychiatry.

[46] Storch E.A., Rasmussen S.A., Price L.H., Larson M.J., Murphy T.K., Goodman W.K. (2010). Development and Psychometric Evaluation of the Yale–Brown Obsessive-Compulsive Scale—Second Edition. Psychol. Assess..

[47] Eisen J.L., Phillips K.A., Baer L., Beer D.A., Atala K.D., Rasmussen S.A. (1998). The Brown Assessment of Beliefs Scale: Reliability and Validity. Am. J. Psychiatry.

[48] Fonagy P., Allison E. (2014). The Role of Mentalizing and Epistemic Trust in the Therapeutic Relationship. Psychotherapy.

[49] Ji Z., Lee N., Frieske R., Yu T., Su D., Xu Y., Ishii E., Bang Y.J., Madotto A., Fung P. (2023). Survey of Hallucination in Natural Language Generation. ACM Comput. Surv..

[50] Zaboski B.A., Therriault D.J. (2020). Faking Science: Scientificness, Credibility, and Belief in Pseudoscience. Educ. Psychol..

[51] Therriault D.J., Zaboski B.A., Jankovsky A. (2022). What Can Fake News, Politics, and Religion Tell Us about Pseudoscience. J. Educ. Soc. Policy.

[52] Risko E.F., Gilbert S.J. (2016). Cognitive Offloading. Trends Cogn. Sci..

[53] Insel T.R. (2017). Digital Phenotyping: Technology for a New Science of Behavior. JAMA.

[54] Kraut R., Patterson M., Lundmark V., Kiesler S., Mukophadhyay T., Scherlis W. (1998). Internet Paradox: A Social Technology That Reduces Social Involvement and Psychological Well-Being?. Am. Psychol..

[55] Kraut R., Kiesler S., Boneva B., Cummings J., Helgeson V., Crawford A. (2002). Internet Paradox Revisited. J. Soc. Issues.

[56] Zaboski B.A., Muller G.N. (2026). The Dual Imperative in AI for OCD: Bridging Ethical Frameworks and Explainable Diagnostics. AI Med..

[57] Hayes S.C. (2022). Acceptance and Defusion. Cogn. Behav. Pract..

[58] Mattera E.F., Ching T.H.W., Zaboski B.A., Kichuk S.A. (2024). Suicidal Obsessions or Suicidal Ideation? A Case Report and Practical Guide for Differential Assessment. Cogn. Behav. Pract..

[59] Mattera E.F., Sandoval K., Sipper C., Fitzpatrick M., Allen A., Pease J., Schild S., Ulaba-Samura Y., Cha C., Pittenger C. (2025). Distinguishing Suicidal Obsessions from Suicidal Ideation: A First-Person Suicide Image Pilot Study. J. Affect. Disord..

[60] Omar M., Sorin V., Collins J.D., Reich D., Freeman R., Gavin N., Charney A., Stump L., Bragazzi N.L., Nadkarni G.N. (2025). Multi-Model Assurance Analysis Showing Large Language Models Are Highly Vulnerable to Adversarial Hallucination Attacks during Clinical Decision Support. Commun. Med..

[61] Yener R., Chen G.-H., Gumusel E., Bashir M. (2025). Can I Trust This Chatbot? Assessing User Privacy in AI -Healthcare Chatbot Applications. Proc. Assoc. Inf. Sci. Technol..

[62] Robins J.M., Hernán M.A., Fitzmaurice G., Davidian M., Verbeke G., Molenberghs G. (2008). Estimation of the Causal Effects of Time-Varying Exposures. Longitudinal Data Analysis.

[63] VanderWeele T.J. (2016). Mediation Analysis: A Practitioner’s Guide. Annu. Rev. Public Health.

[64] Robins J.M., Hernan M.A., Brumback B. (2000). Marginal Structural Models and Causal Inference in Epidemiology. Epidemiology.

[65] Obermeyer Z., Powers B., Vogeli C., Mullainathan S. (2019). Dissecting Racial Bias in an Algorithm Used to Manage the Health of Populations. Science.

[66] Blease C., Lewis M., Riggare S., Fraile Navarro D., Lehman R. (2025). How Generative AI Affects Patient Agency. BMJ.

