# Supplementary material for: The Patient–AI Relationship in Obsessive–Compulsive and Related Disorders: A Cognitive-Behavioral Framework
Source: J Clin Med. 2026 Sep 15;15(18):7154. doi: 10.3390/jcm15187154 (PMC13607497; doi:10.3390/jcm15187154)
Supplement: Supplementary file 1 [file jcm-15-07154-s001.zip › jcm-4543417-supplementary.pdf]

Supplementary Materials

The Patient–AI Relationship in Obsessive-Compulsive and Related Disorders: A Cognitive-Behavioral Framework

Brian A. Zaboski, Emmi Sugino, and Kyle King

S1. Scope and Availability

This supplement documents the development of the Structured Transdiagnostic Assessment of AI Relationships (STaar), the instrument described in Section 4 of the main text. It covers item generation and provenance (Section S2), the mapping of domain facets onto existing instruments (Section S3), derivation of the severity anchors (Section S4), content review to date (Section S5), and the psychometric evaluation now underway (Section S6).

The form itself is not distributed with this Perspective. STaar has not yet been evaluated for factor structure, internal consistency, or inter-rater reliability, and circulating a rating scale before those properties are known invites its use as though they were established. The domains, construct definitions, facets, and representative clinical questions appear in Tables 3 and 4 of the main text, which we judge sufficient for readers to evaluate the construct logic of the framework. The complete form will be released with the psychometric report.

S2. Item Generation and Provenance

Items were generated deductively from the mechanistic account rather than drawn from an existing item pool or selected empirically from a larger candidate set. Three constraints governed generation.

First, each domain had to correspond to a *function* described in Section 3 of the main text rather than to a category or quantity of technology use. Candidate domains that could be satisfied by counting were excluded from scoring on that basis.

Second, facets within each domain were adapted from established instruments rather than written de novo, so that the rating task would be familiar to clinicians already trained in assessments related to the OCRDs. Section S3 sets out the mapping.

Third, no item required the clinician to judge the accuracy of the model's output. The rater assesses the function of the patient's engagement, not the truth of what the system returned. This constraint excluded several candidate items concerning misinformation and hallucination, which we judged to be properties of the product rather than of the patient–AI relationship.

The instrument passed through four drafts. Table S1 records the principal structural changes and what motivated each.

Table S1. Development history of the STaar.

Version	Principal structure	Changes and rationale
1.0	Five domains: Frequency and Context of AI Interaction; Emotional Dependency and Regulation; Anthropomorphic Attachment; Epistemic Trust and Reality Testing; Reassurance and Safety-Seeking. Severity rated 0–4 over a 30-day window.	Initial deductive draft. Four heuristic profile types were drafted alongside the domains (low-risk instrument use; heavy but non-pathological use; an emotion-regulation profile; a reassurance-compulsion profile). These became the phenotypes described in Section 5 of the main text.
2.0	Use context separated into an unscored section.	Frequency and context were removed from scoring because time-

	Behavioral domains given Y-BOCS-style facets and belief domains given BABS-style facets. Displacement added as a scored domain. Adaptive-function and safety adjuncts added. No total score.	based indices conflate load with dysfunction, which is the measurement problem the framework identifies. Displacement was added to capture the continuum from delegated capability to substitution. A total score was rejected because the domains are not commensurable.
2.1	Per-domain interference items removed. Impairment rated once globally in the adjunct section. Domain 3 wording sharpened toward relational role rather than affect.	Content review identified redundancy between per-domain interference ratings and the Displacement construct, and noted that impairment rated within each domain would be counted five times. Anticipated cross-loading between Emotional Reliance and Displacement was registered as a prediction for the analysis plan rather than resolved by revision, on the reasoning that a structure imposed a priori cannot subsequently be tested.
2.2	Current draft. Section A use context, unscored. Domains 1–5: Emotional Reliance, Reassurance and Checking, Anthropomorphic Attribution, Epistemic Trust, Displacement. Section C adjuncts C1 Adaptive Function, C2 Interference and Impairment, C3 Concealment, C4 Safety branch, C5 Product covariates.	Domain labels revised toward function and away from inferred motive. C5 was added after model heterogeneity was identified as a threat to comparability across studies (main text, Section 7). C3 was placed outside the domain profile pending factor analysis rather than assigned to a domain on theoretical grounds.

The form is self-contained. Construct definitions, facets, and anchors are printed on the form, and there is no separate administration manual as rating instructions are included within it.

S3. Facet Provenance and Mapping

Two rating traditions were used. Behavioral domains follow the facet structure of the Yale-Brown Obsessive Compulsive Scale [45,46], in which a behavior is characterized by the time it occupies, the distress associated with its prevention, the patient's resistance to it, and the degree of control retained over it. Belief domains follow the structure of the Brown Assessment of Beliefs Scale [47], in which a belief is characterized by conviction, fixity against counter-evidence, and the behavioral consequences that follow from holding it. The Epistemic Trust construct additionally draws on the account of epistemic trust in the therapeutic relationship given by Fonagy and Allison [48].

Table S2 provides the mapping.

Table S2. Mapping of STaar domain facets to source instruments.

Domain	Type	Facets	Source	Notes
1 · Emotional Reliance (ER)	Behavioral	time occupied; distress on unavailability; resistance; control	Y-BOCS [45, 46]	Direct adaptation. Distress on unavailability substitutes for distress on response prevention, because the relevant contingency is access rather than a therapist-imposed block.
2 · Reassurance and Checking (RC)	Behavioral	time occupied; distress if prevented; resistance; control over the sequence	Y-BOCS [45, 46]	Direct adaptation. Control is specified over the sequence rather than over a single act, because the clinically relevant loss of control concerns repetition and rephrasing rather than the initial query.
3 · Anthropomorphic Attribution (AA)	Belief	conviction; fixity; relational primacy; behavioral consequence	BABS [47]	*Relational primacy* is an extension. It rates the position the system occupies relative to human relationships rather than the strength of belief about its inner life, which conviction and fixity already capture.

4 · Epistemic Trust (ET)	Belief	credibility conviction; fixity against counter-evidence; authority ranking; belief incorporation	BABS [47]; Fonagy and Allison [48]	*Authority ranking* is an extension, rating what the patient does when the system and a human source conflict. It carries the most direct clinical implication.
5 · Displacement (DP)	Behavioral	displacement of human contact; avoidance of unassisted functioning; displacement of other coping and activity; displacement of, or interference with, care	Extension; no source instrument	Facets were written to span the substitution continuum from delegated capability to replaced or countermanded clinical care. Two exclusion criteria are built into the rater notes. Preference for efficiency is not rated. Activity that stopped for unrelated reasons is not rated, which separates displacement from anhedonia.

Each domain is rated from four items scored 0 to 4 and expressed as a mean. Domains are not summed, and any single item rated 3 or above is flagged individually regardless of the domain mean.

S4. Derivation of the Severity Anchors

Anchors run from 0 to 4 and are rated over the preceding 30 days, matching the Y-BOCS rating window and the interval most familiar to clinicians conducting obsessive-compulsive assessment. The rating bands are given in Table S3.

Table S3. Anchor structure.

Rating	Band	Intended meaning
0	Absent or normative	The function is not present, or is present at a level indistinguishable from ordinary use of the tool.
1–2	Subclinical to moderate	The function is present and identifiable. At 1 it is occasional and does not organize behavior. At 2 it recurs and the patient can describe a pattern.
3–4	Severe to extreme	The function organizes behavior. At 3 it is difficult to interrupt. At 4 it is close to continuous, or the patient reports little control over it.

The numeric thresholds were set to distribute plausible clinical presentations across the range rather than derived from a distribution of observed scores (see Section 8 of the main text). Establishing empirically defensible thresholds is an objective of the evaluation described in Section S6.

S5. Content Review to Date

Version 2.0 was circulated to clinical colleagues and individuals with lived experience for content review. Table S4 records the substantive concerns returned and their disposition. No formal content validity index was computed, and the review was structured as expert and lived experience critique rather than as quantified item rating.

Table S4. Content review concerns and dispositions.

Concern raised	Disposition
Construct overlap between Emotional Reliance and Reassurance and Checking.	Construct definitions revised to partition by aim. Emotional Reliance excludes use aimed at settling a question however distressing; Reassurance and Checking excludes use aimed at expression or company. Empirical separation of the two is a hypothesis under test rather than a resolved matter.

Ambiguity in the time-occupied anchors arising from disjunctive conditions.	Anchors rewritten as single-metric hour bands, with episode frequency recorded in the notes rather than folded into the rating (Section S4).
Domain 2 construct framed in terms specific to obsessive-compulsive disorder, limiting transdiagnostic use.	Wording generalized from compulsion-specific language to the reduction of uncertainty and the neutralization of a feared outcome. This covers appearance-related checking in body dysmorphic disorder and health-related checking without presupposing an obsessional structure.
Placement of the concealment rating within the domain profile.	Concealment was moved outside the profile and is rated as adjunct C3 pending factor analysis, on the basis that it may prove to be a severity marker cross-cutting the domains rather than a feature of any one of them.
Philosophical wording in the Domain 4 construct definition.	Revised toward observable behavior. The domain now rates what the patient treats as authoritative and what they do when sources conflict, rather than the epistemological status they assign to machine-generated claims.
Per-domain interference items redundant with Displacement and with global impairment.	Removed in version 2.1. Impairment is rated once globally as adjunct C2.

S6. Psychometric Evaluation

The evaluation now underway addresses three objectives.

Objective 1. Item performance and preliminary validity. Item-level discrimination and distributional properties across the five domains, with preliminary convergent and discriminant validity against established measures. Convergent expectations are strongest for Reassurance and Checking against obsessive-compulsive severity. Discriminant expectations are strongest for Emotional Reliance against Displacement, which is the cross-loading registered in Section S2.

Objective 2. Inter-rater reliability. Rater calibration on standardized vignettes prior to live administration, with double coding of live interviews and adjudication of discrepancies. Calibration will require a prespecified agreement threshold before live rating proceeds.

Objective 3. Phenotype recovery. Whether the profiles hypothesized in Section 5 of the main text are recoverable from observed domain configurations, or whether the data support a different structure. This includes the possibility that Displacement functions as a severity gradient cross-cutting the other four domains. The objective is explicitly capable of disconfirming the phenotype account.
